# Detailed morphological structure and phylogenetic relationships of *Degeeriella punctifer* (Phthiraptera: Philopteridae), a parasite of the bearded vulture *Gypaetus barbatus* (Accipitriformes: Accipitridae)

**DOI:** 10.1038/s41598-023-27774-2

**Published:** 2023-01-10

**Authors:** Jesús M. Pérez, Ángeles Sáez-Ventura, Gracia Liébanas, Luca Rossi, Mercedes Fernández, Natalia Fraija-Fernández

**Affiliations:** 1 Departamento de Biología Animal, Biología Vegetal y Ecología, Universidad de Jaén, Campus Las Lagunillas, s/n, 23071 Jaén, Spain; 2 Wildlife Ecology and Health (WE&H) Group, Barcelona, Spain; 3 Dipartimento di Science Veterinarie, Università di Torino, Largo Paolo Braccini 2, 10095 Grugliasco, Torino, Italy; 4 Instituto Cavanilles de Biodiversidad y Biología Evolutiva (ICBiBE), Universidad de Valencia, Catedrático José Beltrán, nº 2, 46980 Paterna, Valencia Spain

**Keywords:** Ecology, Genetics, Zoology

## Abstract

Habitat loss is one of the main threats to species survival and, in the case of parasites, it is their hosts that provide their habitat. Therefore, extinction even at local scale of host taxa also implies the extinction of their parasites in a process known as co-extinction. This is the case of the bearded vulture (*Gypaetus barbatus*), which almost became extinct at the beginning of the twentieth century. After several attempts, this species was successfully reintroduced into the Alps at the end of the twentieth century. We collected 25 lice specimens from an electrocuted bearded vulture from Susa (Italian Alps) that were morphologically identified as *Degeeriella punctifer*. Six individuals were studied by scanning electron microscopy, with particular emphasis on their cephalic sensorial structures, while four further specimens were characterized at molecular level by amplifying partial regions of the *12SrRNA*, *COX1* and elongation factor 1 alpha (*EF*-1) genes. From a morphological perspective, the number, type and arrangement of the sensillae on the two distal antennal segments is quite similar to that of other species of the family Philopteridae (Phthiraptera: Ischnocera). The mandibles and tarsal claws allow lice to cling firmly to their host’s feathers. Phylogenetic analyses help unravel the paraphyletic nature of the genus *Degeeriella* and demonstrate the clear differentiation between lice parasitizing Accipitriformes and Falconiformes, as well as the close relationship between *D. punctifer, D. fulva, D. nisus* and *Capraiella* sp. that, along with other genera, parasitize rollers (Aves: Coraciiformes).

## Introduction

Lice (order Phthiraptera) are ectoparasitic insects that infest birds and mammals. They exhibit a certain degree of host specificity to the extent that many lice species only occur on a single host species^1^. As available hosts represent suitable habitat for their parasites, we can expect that the potential distribution range of each louse species matches the distribution range of its host. Therefore, the extinction of a host species will lead to the extinction of the lice it harbours, a process known as co-extinction^2,3^, both at global and local levels. Within this context, we can thus expect there to be a parallelism between the conservation status of a host and its ectoparasites. As an example, both the sucking louse *Haematopinus oliveri* and the pygmy hog *Porcula salvinia*, its only known host, are catalogued as critically endangered^4^.

The bearded vulture (*Gypaetus barbatus*) died out in the Alps in the early twentieth century, mainly as a result of human persecution. In 1986 a reintroduction program was initiated, based on the release of captive-bred young individuals brought mainly from the Pyrenees. The first successful breeding event in the wild for this re-established Alpine population was reported in 1997^5,6^. Currently, both at global and European levels, this species is catalogued as Near Threatened (NT) and has a negative trend^7^.

This vulture is parasitized by several species of ectoparasites, which are affected by the same pressures as threaten their host. Four lice species have been found on bearded vultures: *Degeeriella punctifer* (Gervais 1844) and *Falcolipeurus quadripustulatus* (Giebel 1861) (Ischnocera, Philopteridae), *Colpocephalum barbati* Price and Beer 1963 (Amblycera, Menoponidae), and *Laemobothrion (Laemobothrion) vulturis* (Fabricius 1775) (Amblycera, Laemobothriidae). To the best of our knowledge, *D. punctifer* and *C. barbati* only parasitize the bearded vulture^8^. Therefore, the reintroduction of the bearded vulture in the Alps described above was an excellent opportunity to test how keeping this bird captivity affects its ectoparasites.

Taxonomically, species of the genera *Degeeriella*, *Acutifrons*, *Austrophilopterus*, *Capraiella*, *Cotingacola*, *Cuculicola*, *Lagopecus*, *Picicola* and *Trogonirmus*, among others, were grouped within the *Degeeriella* complex based on certain difficult-to-classify morphological characteristics^9^. Martín Mateo^10^ also considers the species from this complex to be difficult to classify given their morphological resemblance. Thus, their taxonomy is controversial. In general, feather lice—specifically, species from the *Degeeriella* complex—have a certain degree of higher-level classification correspondence with their avian hosts (e.g. Order)^11^. Several authors^12,13^ consider the *Degeeriella* complex and the genus *Degeeriella* to be paraphyletic, with lice from Falconiformes (e.g. *D. carruthi* and *D. rufa*) and Accipitriformes (e.g. *D. fulva*, *D. fusca*, *D. frater*, *D. haydocki*, *D. nisus*, *D. quatei*, *D. regalis*, *D. rima*, *D. vagans* and *Capraiella* spp.) forming two distinct clades^13^.

*Degeeriella* is one of the most frequently found lice genera on raptors^14^. Ischnoceran lice, as is the case of *Degeeriella* spp., feed on a hosts’ feathers and are more host-specific and restricted to particular regions of the host’s body than Amblycera lice^15–17^. Indeed, *Gypaetus barbatus* is the only known host species of *D. punctifer*^8,9^.

In recent decades, morphological research using Scanning Electron Microscopy (SEM), higher magnification and 3D visualisation have facilitated detailed morphological studies of lice. Results obtained with SEM have proved to be useful not only for confirming the taxonomic identity of specimens but also for addressing research subjects including the phoretic behaviour of Ischnoceran and Amblyceran lice^18^, the adaptation of lice to aquatic life^19^, and the function of different antennal receptors^20,21^.

Simultaneously, in this period, molecular genetic studies have updated the phylogenetic relationships between Phthiraptera, previously only inferred from morpho-biometric data. For instance, the mitochondrial cytochrome b has been used to characterize host-parasite cospeciation events^22^ and partial sequences of the elongation factor 1 alpha have shed light on the identification of major groups of lice^23^. Based on the small subunit of ribosomal RNA, Murrel and Barker^24^ provide evidence of the paraphyletic origin of Phthiraptera, while microevolutionary studies of multiple parasites on a single host have been carried out based on homologous regions of mitochondrial cytochrome oxidase I and mitochondrial small subunit ribosomal RNA^25^.

Access to lice specimens collected from an electrocuted bearded vulture from the Italian Alps allowed us to study the complex taxonomy and phylogeny of *Degeeriella.* This study aimed to: (1) confirm the identification at morphological and molecular level of the feather lice found on this bearded vulture; (2) provide a detailed 3D morphological description of the lice specimens to complement a previous description by^9^; and (3) determine the phylogenetic position of the collected lice species and their relationship with other feather lice taxa.

## Material and methods

### Sample collection

A total of 25 lice were collected in April 2018 from a fresh carcass of an adult female bearded vulture from Susa (Italian Alps) (45°18′57.28″ N–7°02′72.16″ E), found dead after a collision with a power line. After collection, lice were fixed in 70% ethanol until processing in the laboratory.

### Morphological analyses

Eight individuals (four males, three females and one nymph) were mounted in Canada balsam following^26^ for observation using light microscopy. The remaining specimens were temporarily mounted in water to obtain measurements. Measurements (in µm) of the length and width of head, thorax, abdomen and parameres of males from each specimen were taken using a Nikon Eclipse 80i optical microscope (Nikon, Tokyo, Japan) equipped with a differential optical camera with optical inference contrast (DIC), and a Nikon Digital Sight DS-U1 digital camera. Cephalic (head width/head length) and body (maximum width/total length) indices were also obtained for each specimen. A further six specimens (three males, two females and one nymph) were selected for observation using SEM. To eliminate particles adhered to the cuticle, specimens were placed in a solution of 70% ethanol and 30% ether for three days^27^. Subsequently, specimens were submitted to an ultrasound session lasting 20 min with a medium emission frequency at 30% intensity. Individuals were cleaned with distilled water and dehydrated with a series of increasing concentrations of ethanol, finishing in acetone. A critical point was obtained before gold coating. Observations were made using a Zeiss Merlin scanning electron microscope (Carl Zeiss, Germany).

### DNA extraction and amplification

The genomic DNA of four lice was extracted using the DNeasy Blood and Tissue extraction kit (QIAGEN, Hilden, Germany) following the manufacturer’s instructions in all except for the initial incubation time, which was set to 48 h, and the final elution volume, established as 100 μL. The mitochondrial cytochrome oxidase I (*cox1*), mitochondrial small subunit ribosomal RNA (*12S rRNA gene*), and the nuclear elongation factor 1 alpha (*EF-1*) were amplified (Table 2) using the following set of primers: (a) L6625 (5′-CCGGATCCTTYTGRTTYTTYGGNCAYCC-3′) and H7005 (5′-CCGGATCCACNACRTARTANGTRTCRTG-3′) for *cox1*^28^; (b) 12Sai (5′-AAACTAGGATTAGATACCCTATTAT-3′) and 12Sbi (5′-AAGAGCGACGGGCGATGTGT-3′) for *12SrRNA*^29^; and (c) EF1-For3 (5′-GGNGACAAYGTTGGYTTCAACG-3′) and Cho10 (5′-ACRGCVACKGTYTGHCKCATGTC-3′) for *EF-1*^30^. The final volume for each PCR was 20 µL, including 2 µL of DNA, 4.8 µL of water, 1.6 µL of each primer, and 10 µL of DNA Polymerase MyFi (BioLine, Meridian Life Science Inc., Tauton, USA). In all cases, an additional sample without DNA was included as a negative control. The thermocycling profile was performed under the conditions described by Smith et al.^31^. PCR products were observed on a 1% agarose gel and, in cases of poor amplification, a second PCR was performed with a greater number of cycles and greater alignment time. The amplified material was then purified with the Nucleospin PCR and Gel Purification Clean-up kit (Machery-Nagel, Düren, Germany) and sent to Macrogen (Spain) for sequencing. Sequences were trimmed for low-quality reads and assembled in Geneious Prime 2019.2.1 (https://www.geneious.com). To confirm the specific identity, obtained sequences were compared with the NCBI database using BLAST (Basic Local Alignment Search Tool)^32^.

### Phylogenetic analyses

The obtained sequences were aligned independently for each gene using the online version of Mafft (https://mafft.cbrc.jp/alignment/server/) and comparing with other Philopteridae sequences retrieved from GenBank (Table 1). The nucleotide alignment of the protein-coding gene (*cox1*) was edited manually, in frame, using Geneious. Four datasets were analysed: (i–iii) each gene independently and (iv) concatenated sequences for all three genes. Evolutionary models for each gene were chosen using the *jModelTest2* program^33^ and phylogenetic inferences were obtained using Bayesian Inference (BI) and Maximum Likelihood (ML) analyses. BI was performed using *MrBayes*^34^ and run for 1,000,000 generations; burn-in was set at the point at which the average standard deviation split frequencies was < 0.01. ML analysis was obtained using GARLI (Genetic Algorithm for Rapid Likelihood Inference^35^ and support values for each node were obtained after 100 replications. The obtained phylogenetic trees were manually edited with *FigTree* 1.4.2^36^. Clades were considered to have high nodal support if BI pp was > 0.8 and ML bootstrap values > 70%.

**Table 1:** GenBank accession numbers of the *12S rRNA*, *cox1* and *EF-1* sequences analysed in this study.

Species	Host order	GenBank accession numbers			Dataset			
		cox1	12S rRNA	EF-1				
**OUTGROUP**								
*Brueelia* sp.			AY314850			Dataset 2		
*Chelopistes texanus*		AF348857		AF447191	Dataset 1		Dataset 3	Dataset 4
*Quadraceps punctatus*		AF348864	AF396526		Dataset 1	Dataset 2		Dataset 4
**INGROUP**								
*Austrophilopterus pacificus*		AF444846		AF447184	Dataset 1		Dataset 3	Dataset 4
*Austrophilopterus subsimilis*		AF385001	AF189130		Dataset 1	Dataset 2		Dataset 4
*Austrophilopterus torquatus*		AF444849		AF447187			Dataset 3	Dataset 4
*Capraiella* sp.		MG682408		AF447190	Dataset 1		Dataset 3	Dataset 4
*Cotingacola stotzi*		AF444854		AF447192	Dataset 1		Dataset 3	Dataset 4
*Cuclotogaster hopkinsi*		AF348875	AY314854		Dataset 1	Dataset 2		Dataset 4
*Degeeriella carruthi*		AY314813			Dataset 1			
*Degeeriella carruthi*		AF444860		AF447196	Dataset 1		Dataset 3	Dataset 4
*Degeeriella fulva*	Accipitriformes	AF444861			Dataset 1			
*Degeeriella fulva*	Accipitriformes	KX865181	KX865253	AF447197	Dataset 1	Dataset 2	Dataset 3	Dataset 4
*Degeeriella nisus*	Accipitriformes	KX865178	KX865249		Dataset 1	Dataset 2		Dataset 4
***Degeeriella punctifer***	Accipitriformes	**[ON171831]**	**[ON171827]**		Dataset 1	Dataset 2		Dataset 4
***Degeeriella punctifer***	Accipitriformes	**[ON171832]**	**[ON171828]**	**[ON171835]**	Dataset 1	Dataset 2	Dataset 3	Dataset 4
***Degeeriella punctifer***	Accipitriformes	**[ON171833]**	**[ON171829]**		Dataset 1	Dataset 2		Dataset 4
***Degeeriella punctifer***	Accipitriformes	**[ON171834]**	**[ON171830]**	**[ON171836]**	Dataset 1	Dataset 2	Dataset 3	Dataset 4
*Degeeriella regalis*	Accipitriformes		DQ490701			Dataset 2		Dataset 4
*Degeeriella rufa*	Falconiformes		AY314855	AY314831		Dataset 2	Dataset 3	Dataset 4
*Picicola capitatus*		AF444866		AF447201	Dataset 1		Dataset 3	Dataset 4
*Picicola porisma*		AF444867		AF447202	Dataset 1		Dataset 3	Dataset 4
*Picicola snodgrassi*		AF444868		AF447203	Dataset 1		Dataset 3	Dataset 4
*Picicola* sp.		KU187326		AF447208	Dataset 1		Dataset 3	Dataset 4

The datasets used for the phylogenetic analyses performed in this study are specified. Newly obtained sequences for *Degeeriella punctifer* are highlighted in bold.

## Results

### Morphological analyses

**Figure 1:**
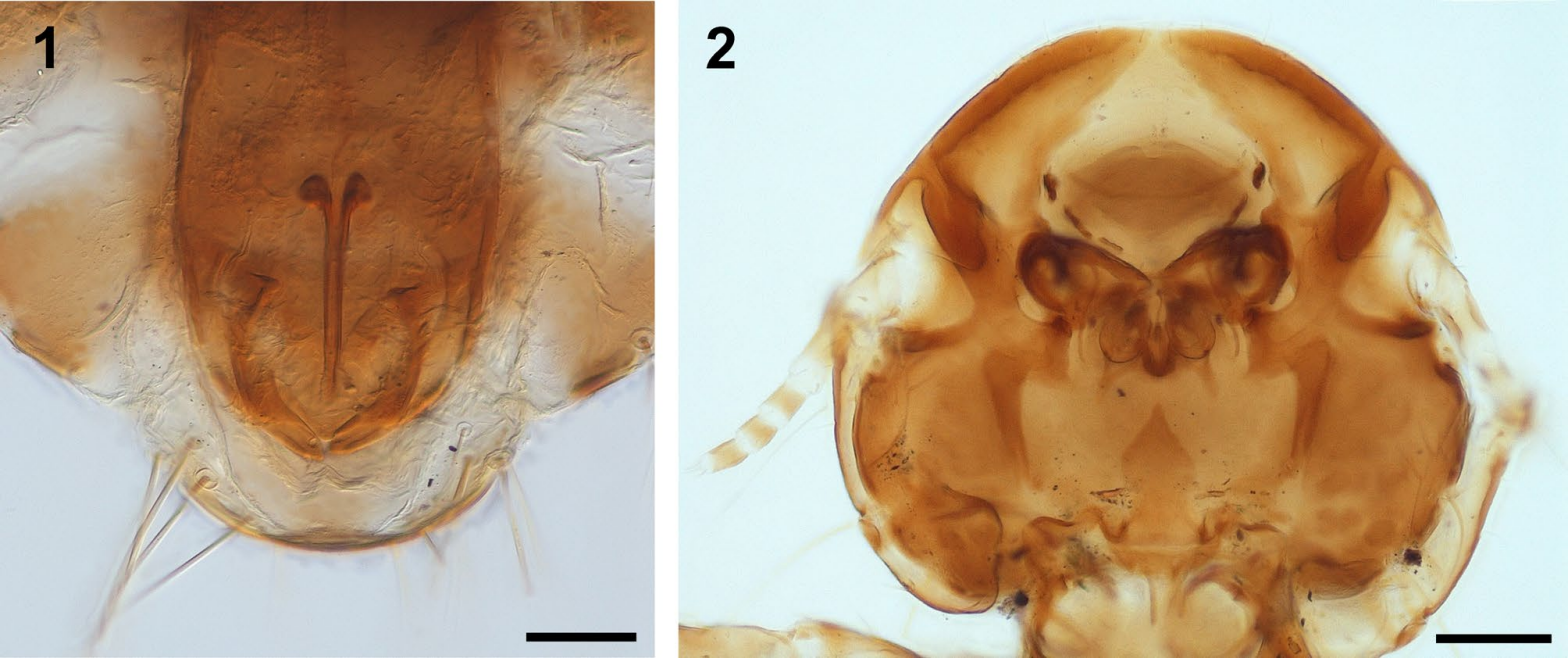
**(1)** *Degeeriella punctifer*. Male genitalia. Scale bar: 100 µm, (**2**) *Degeeriella punctifer*. Head shape. Scale bar: 100 µm.

Lice were morphologically identified as *Degeeriella punctifer* (Gervais, 1758) (Ischnocera: Philopteridae). This species is included in the *phlyctopygus* group^9^, which is characterized by a distinctive type of genitalia with penial sclerite (Fig. 1) and, normally, more than four sternocentral silks in segments III and IV. The following description of adult males and females, and measurements of nymphs, is based on the examination of specimens with optical and scanning electron microscopy.

**Figure 2:**
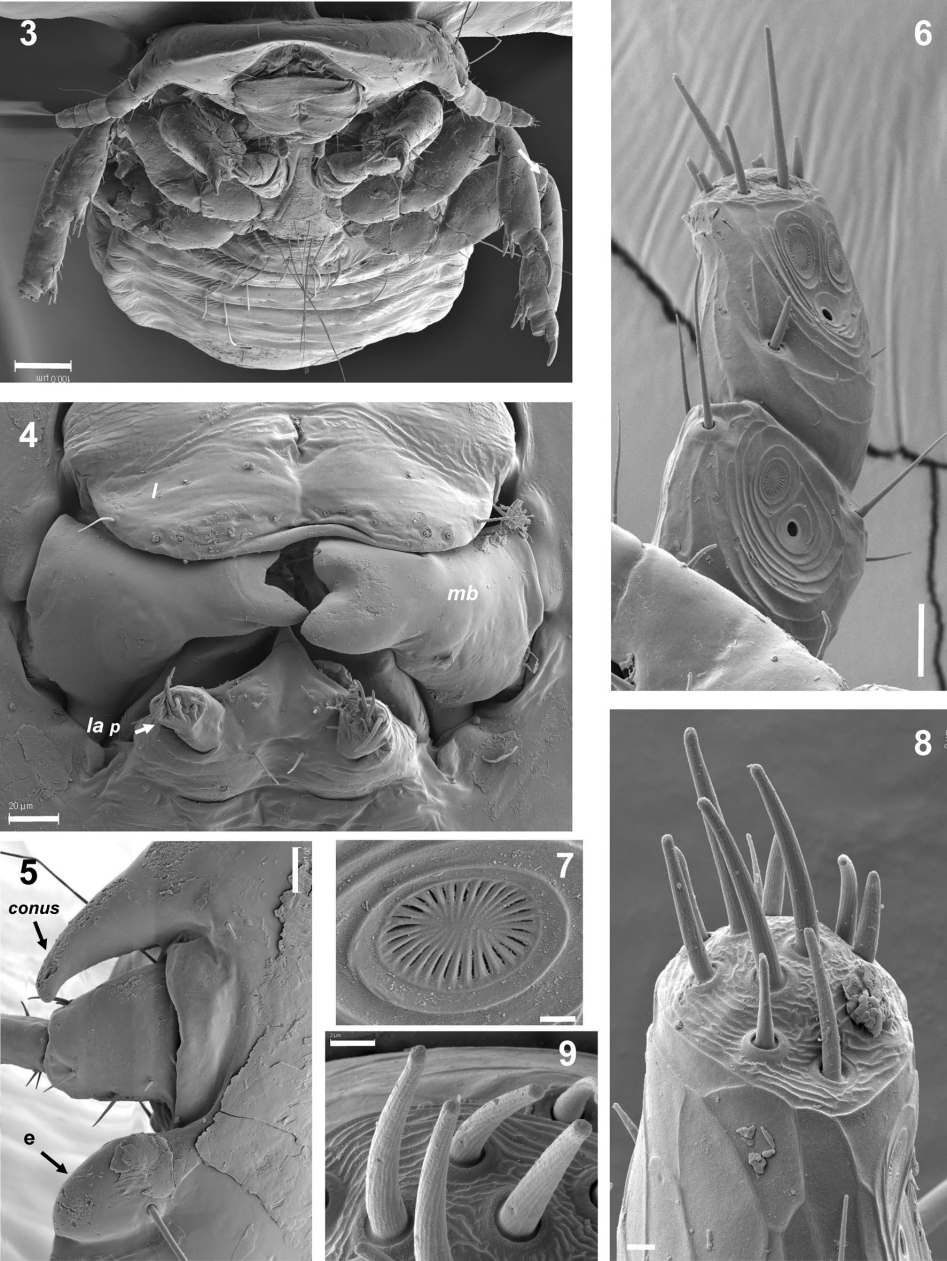
**(3)** *Degeeriella punctifer*. Frontal view of the head. Scale bar: 100 µm. **(4)** *Degeeriella punctifer*. Mandibles and labial palpi. Scale bar: 20 µm. **(5)** *Degeeriella punctifer*. Conus, antennal insertion and eye. Scale bar: 20 µm. **(6)** *Degeeriella punctifer*. Sensillae of the two distal segments of the antenna. Scale bar: 10 µm. **(7)** *Degeeriella punctifer*. Detail of a placoid sensilla. Scale bar: 1 µm. **(8)** *Degeeriella punctifer*. Detail of the tip of the antenna. Scale bar: 2 µm. **(9)** *Degeeriella punctifer*. Detail of the basiconic sensillae of the tip of the antenna. Scale bar: 2 µm.

**Figure 3:**
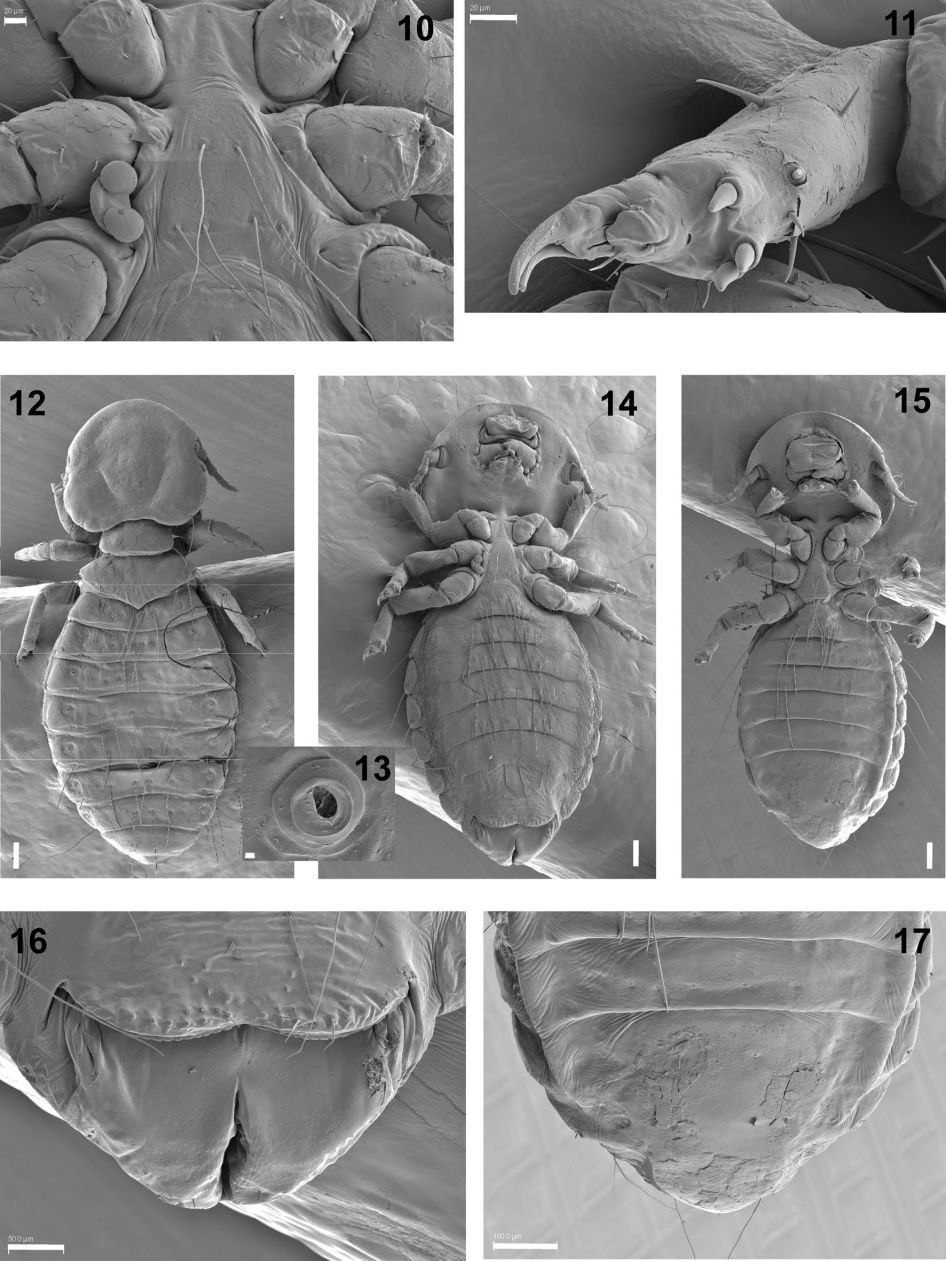
**(10)** *Degeeriella punctifer*. Sternal thoracic plate**.** Scale bar: 20 µm. **(11)** *Degeeriella punctifer*. Tarsal claws and tibial processes. Scale bar: 20 µm. **(12)** *Degeeriella punctifer*. Dorsal view of an adult female showing the arrangement of the spiracles. Scale bar: 100 µm. **(13)** *Degeeriella punctifer*. Detail of a spiracle. Scale bar: 2 µm. **(14)** *Degeeriella punctifer*. Ventral view of an adult female. Scale bar: 100 µm. **(15)** *Degeeriella punctifer*. Ventral view of an adult male. Scale bar: 100 µm. **(16)** *Degeeriella punctifer*. Female. Tip of the abdomen. Scale bar: 50 µm. **(17)** *Degeeriella punctifer*. Male. Tip of the abdomen. Scale bar: 100 µm.

Males (n = 9). Head rounded with the marginal carina ventrally thinned anteriorly, with a narrow hyaline margin and a postantenal suture present (Figs. 1(2)–2(3)). The cephalic index is > 0.94 (Table 2). Mandibles are large and strongly sclerotized while the labial palps are short and unisegmented, with six basiconic setae at the apex (Fig. 2(4)). The coni, which are lateral extensions of the head, lie just above the articulations of the antennae; the small simple eyes are elongated (Fig. 2(5)). The antennae are composed of five segments, with a distinct arrangement of pores and placoid sensillae on the inner side of the final two segments and basiconic sensillae at the apex (Fig. 2(6–9)). The sternal thoracic plate is subtriangular, with two anterior setae and four setae on the posterior margin (Fig. 3(10)). The legs are robust and have three tibial processes, with two tarsal claws (Fig. 3(11)). The spiracles are located on the lateral side of terguites II–VII (Fig. 3(12,13)). Tergum II has an unscletorized central area and tergum III medial narrowing; the remaining terga are elongated and cover the entire width of the abdomen^9^, which give this species a distinctive dorsal appearance. Four setae are inserted into the genital plate, as described by^9^.

**Table 2:** Biometrical data obtained from the *Degeeriella punctifer* specimens analysed in this study.

	Males (n = 9)				Females (n = 3)				Nymphs (n = 7)			
	$\overline{X}$ ± *SD*	Min	Max	CI (95%)	$\overline{X}$ ± *SD*	Min	Max	CI (95%)	$\overline{X}$ ± *SD*	Min	Max	CI (95%)
HL	484.3 ± 21.1	452.6	511.0	472.4–496.2	528.9 ± 20.6	510.2	562.3	510.9–546.9	431.8 ± 58.2	368.1	553.0	391.4–472.2
HW	608.5 ± 15.0	581.8	644.6	600.1–617.0	661.6 ± 17.9	641.7	688.1	645.9–677.3	528.3 ± 62.2	444.1	661.2	485.3–571.4
CeI	1.26 ± 0.05	1.19	1.34	1.23–1.29	1.26 ± 0.05	1.19	1.34	1.20–1.30	1.23 ± 0.05	1.13	1.31	1.19–1.26
TL	360.2 ± 42.2	304.2	463.0	336.3–384.0	451.5 ± 81.1	350.4	526.5	380.4–522.6	315.5 ± 36.0	260.7	373.7	290.6–340.5
TW	457.2 ± 38.7	372.1	489.9	435.4–479.1	550.3 ± 26.5	523.9	592.5	527.0–573.5	426.7 ± 63.0	328.3	527.0	383.0–470.3
AL	1063.1 ± 120.9	883.5	1265.9	994.7–1131.5	1288.6 ± 210.9	1055.5	1613.9	1103.7–1473.4	948.8 ± 150.6	746.4	1152.9	844.4–1053.2
AW	680.9 ± 143.0	362.5	850.3	600.0–761.8	817.6 ± 126.1	613.6	954.5	707.1–928.2	679.4 ± 100.9	537.8	816.5	609.4–749.3
ToL	1907.6 ± 130.4	1680.3	2083.0	1833.8–1981.3	2271.1 ± 182.7	2028.3	2482.3	2111.0–2431.3	1696.1 ± 199.1	1375.2	2018.0	1558.2–1834.1
CoI	0.37 ± 0.03	0.33	0.43	0.35–0.39	0.36 ± 0.03	0.32	0.38	0.34–0.38	0.39 ± 0.02	0.37	0.44	0.38–0.42
PaL	94.2 ± 12.3	78.1	115.9	87.2–101.2	NA	NA	NA	NA	NA	NA	NA	NA

HL: head length; HW: head width; CeI: cephalic index; TL: thoracic length; TW: thoracic width; AL: abdominal length; AW: abdominal width; ToL: total length; CoI: corporal index; PaL: parameres length. Measurements are shown in micrometers and expressed as the mean ($\overline{X}$) ± standard deviation ($SD$), followed by the minimum (Min) and maximum (Max) values, and the confidence interval (CI) at 95%. n: number of specimens analysed. NA: not applicable.

Females (n = 3). Their appearance is similar to that of males, except for body size as the females are larger than the males, above all in the abdomen (Fig. 3(14,15); Table 2). The terga of segments IX–XI and genital region are as described by Clay (1958). The chaetotaxy of both sexes coincides with^9^ Clay’s description (1958) for *D. punctifer*. The terminal part of the abdomen at ventral level in females is quite distinct from that of the males, and has a longitudinal genital opening (Fig. 3(16,17)).

Nymphs (n = 7): specimens were smaller in length than adults (Table 2) and less chitinized, which gives them a more transparent appearance.

### Molecular and phylogenetic analyses

Four sequences of *D. punctifer* were obtained for the *12S rRNA* and *cox1* genes, with lengths varying between 564 and 596 bp, and between 436 and 437 bp, respectively. Additionally, we obtained two sequences for the *EF-1* gene of *D. punctifer* with 360 bp each (Table 1). The nucleotide similarity of the obtained sequences reached 99.5%, 98.6% and 90.4% for the *12S rRNA, cox1* and *EF-1* sequences, respectively.

**Figure 4:**
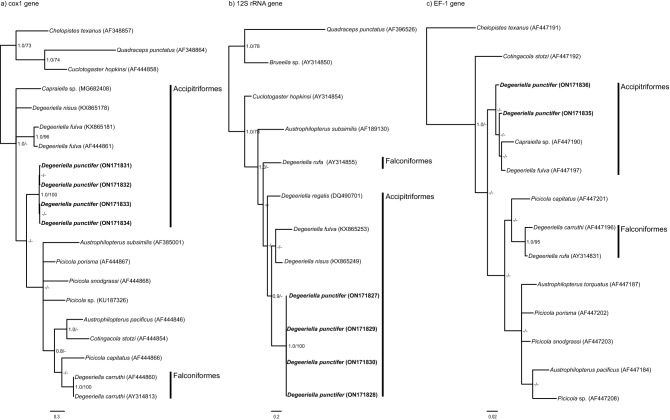
Phylogenetic tree depicting the relationships of *Degeriella punctifer* based on the Bayesian analysis of the (**a**) *cox1*, (**b**) *12S rRNA* and (**c**) *EF1* genes analysed independently, and constructed using *MrBayes* v3.2.2 under the GTR+I+G model of nucleotide evolution for each gene. Posterior probabilities and maximum likelihood bootstrap support values (100 replicates) are given for each node. Posterior probabilities < 0.8 and bootstrap support values < 70% are not shown. The scale bars indicate the number of substitutions per site. The host order is given for *Degeeriella* species occurring in Falconiformes and Accipitriformes.

**Figure 5:**
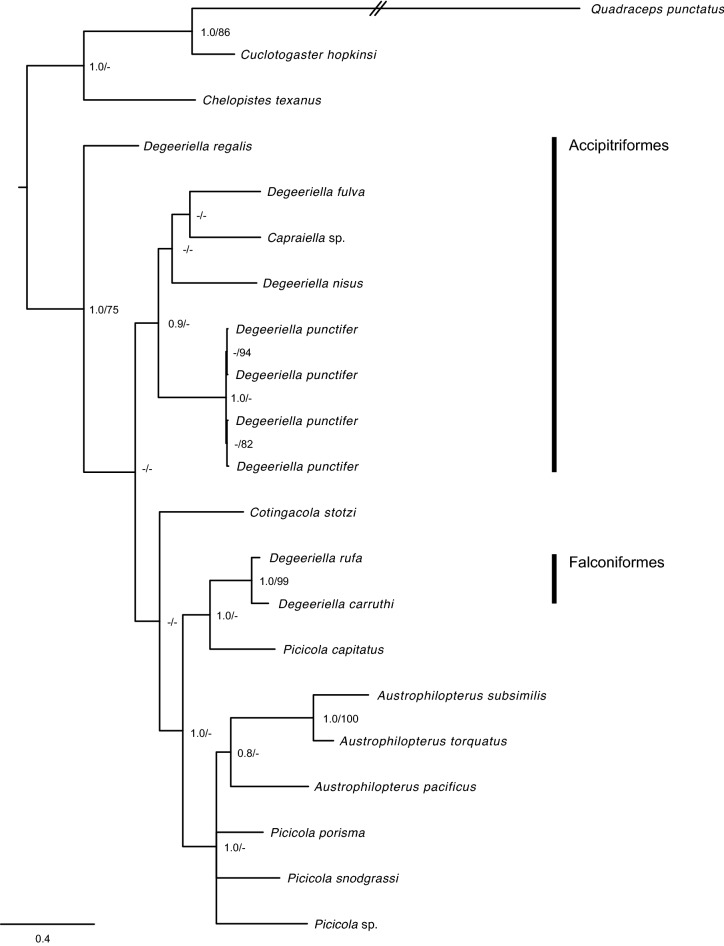
Phylogenetic tree depicting the relationships of *Degeeriella punctifer* based on the Bayesian analysis of the concatenated matrix (*cox1* + *12S rRNA* + *EF1* genes), constructed using *MrBayes* v3.2.2 under the GTR+I+G model of nucleotide evolution for each partition. Posterior probabilities and maximum likelihood bootstrap support values (100 replicates) are given for each node. Posterior probabilities < 0.8 and bootstrap support values < 70% are not shown. The scale bars indicate the number of substitutions per site. The host order is given for *Degeeriella* species occurring in Falconiformes and Accipitriformes.

Datasets 1–3 for single gene analyses included 12, 20 and 14 sequences for the *12S rRNA*, *cox1* and *EF-1* genes, respectively, whereas the concatenated analysis for the three genes included a total of 21 sequences (Table 1). All datasets rendered the *Degeeriella* complex as paraphyletic (Fig. 5). All newly obtained sequences for *D. punctifer* were grouped in a monophyletic clade, except when the *EF-1* gene was analysed independently (Dataset 3) (Fig. 4c). A clade formed by *D. fulva*, *Capraiella* sp. and *D. nisus* was consistently placed as sister to *D. punctifer* when the three concatenated genes were analysed, although it was only well supported by Bayesian posterior probabilities but not by ML bootstrap values (MB pp = 0.9; ML bootstrap = 42%) (Fig. 5). A similar relationship between *D. punctifer, D. fulva* and *Capraiella* sp. was observed when the *12S rRNA* and the *EF-1* genes were analysed independently, but not for the *cox 1* gene from which the phylogenetic relationship could not be clearly identified (Fig. 4). Of the *Degeeriella* species occurring on Accipitriformes, *D. regalis* occupies an early-diverging position (Figs. 4b, 5). The analysed *Degeeriella* species occurring on Falconiformes (i.e. *D. rufa* and *D. carrutthi*) were consistently placed as sisters to *Picicola capitatus* (Fig. 4c). The other *Picicola* spp. analysed in this study were closely related to *Austrophilopterus* spp., although the phylogenetic relationship for these species could not be clearly ascertained (Figs. 4, 5).

## Discussion

This study reports for the first time the occurrence of *D. punctifer* in the Italian Alps having been reported previously from Afghanistan and Sikkim^9^, Lesotho^37^, Spain^10^ and the Indian Himalayas^38^. The size of our specimens falls into the size range reported by Clay^9^ for this lice species.

In lice, the antennae are the main peripheral sensory organs^20^. The structure, morphology and function of antennae in Phthirapteran suborders and families are very similar in all species and follow a common pattern^21,39^. In fact, the shape, number and distribution pattern of sensillae at the distal tip of the antennae of *D. punctifer* correspond to those described for *D. fulva*, *D. regalis*, *Craspedorrhynchus platystomus*, *Bueelia* spp. and *Upupicola upupae*^40–43^.

One remarkable characteristic of the specimens of *D. punctifer* analysed in this study was the sturdy composition of the mandibles and tarsal claws (Figs. 2(4), 3(11)). Ischnoceran lice use their mandibles and claws, as observed in *D. punctifer*, for attaching themselves firmly to host feathers or to phoretic flies (e.g. hippoboscids) but compared to Amblyceran lice move around little when not on their hosts^18^.

The immature specimens studied were quite similar to others in terms of both morphology and size, and were classified as third-instar nymphs. Nevertheless, the first- and second-instar nymphs of *D. punctifer* are still unknown, so the morphological description of all developmental stages of this species remains incomplete^44^.

We provide here for the first time the molecular sequences of partial *cox1*, *12SrRNA* and *EF-1* of *D. punctifer*. From the phylogenetic relationships analysed in this study, the sequences of each of the genes obtained were grouped together in a monophyletic clade, the exception being the *EF-1* tree (Fig. 4c). Even though the two *EF-1* sequences of *D. punctifer* were the same length (360 bp), there were many more ambiguities regarding specific nucleotide positions in one of the sequences, indicated by the low percentage of similarity between them (90.1%). Nevertheless, the monophyletic and well-supported clade (Bayesian pp = 1.0 and ML bootstrap = 100%) formed by the four sequences obtained for both mitochondrial genes (*cox1* and *12SrRNA*) is evidence that the four specimens used for the molecular analyses correspond to the same specific entity. Furthermore, a monophyletic clade formed by *D. punctifer, D. fulva* and *Capraiella* sp. was consistent when the *EF-1* gene was analysed independently and when the three genes were concatenated (Fig. 5).

In general, *D. punctifer* was found to be closely related to a clade consisting of *D. fulva* and *Capraiella* sp. + *D. nisus* (Fig. 5), all of which are parasites of Accipitriformes. The distant phylogenetic position of *D. regalis*, also present on Accipitriformes, and *D. rufa* and *D. carruthi* on Falconiformes, demonstrates that the genera *Degeeriella* is paraphyletic, as suggested by previous molecular studies. Johnson et al.^12^ used a more extensive phylogenetic context, including other species from the *Degeeriella* complex but only *cox1* and *EF-1* sequences, to show a sister relationship between *D. carruthi* and *Picicola* spp. from African woodpeckers, and *D. fulva* and *Capraiella* sp. from rollers (Coraciidae). In addition, Catanach and Johnson^13^ also extensively studied the phylogenetic relationships of *Degeeriella* spp. and show it to be a paraphyletic genus, with a significant geographical structure for a parasite species that broadly reflects the higher taxonomic level distribution (i.e. Order) of its hosts^13^. These studies, together with the phylogenetic context for *D. punctifer* provided in this study, suggest the need for a re-organization of the taxonomy of *Degeeriella*, *Capraiella* and *Piciola* species as the current taxonomy does not reflect their evolutionary relationships^12^.

Some but not all lice genera show strong evidence of cospeciation and this may be due—at least in part—to differences in their dispersive abilities^45^. This needs be tested within the genus *Degeeriella*, which includes species like *D. fulva* and *D. rufa* that have a wide range of host species. Nevertheless, we lack molecular data for > 60% of the *Degeeriella* taxa. Therefore, further information is needed if we are to perform a reconciliation analysis of the phylogenies of *Degeeriella* species and their respective hosts to characterize cospeciation, host switching, extinction or other macroevolutionary events that may affect these species^25,45–49^.

Of the four lice species parasitizing the bearded vulture, we only found specimens of *D. punctifer*. Ischnoceran lice have a greater capacity to remain on host feathers than Amblyceran lice^18^, although in our case no *Falcolipeurus quadripustulatus* specimens were found. The reintroduced bearded vultures were bred and kept in captivity^50^ and so it seems that these conditions did not negatively influence the survival of ectoparasitic lice on these captive birds^51^. Further studies are still required to detect whether or not the same situation occurs with the other lice species that parasitize the bearded vulture. Finally, if the term co-extinction was coined to describe the extinction of a host and its ectoparasitic lice^2,3^, then we believe it to be logical to talk about the ‘co-reintroduction’ or ‘co-recovery’ of a bird host and, at least, one of its ectoparasitic lice species.

## Data Availability

The datasets generated and/or analysed during the current study are available as Supplementary Data, and in the GenBank® repository at NCBI under accession numbers [ON171827, ON171828, ON171829 and ON171830] (*12S rRNA*), [ON171831, ON171832, ON171833 and ON171834] (*cox1*), and [ON171835 and ON171836] (*EF-1*).

## References

[1] Durden L, Mullen G, Durden L (2019). Lice (Phthiraptera). Medical and Veterinary Entomology.

[2] Stork NE, Lyal CHC (1993). Extinction or ‘co-extinction’ rates?. Nature.

[3] Koh LP, Dunn RR, Sodhi NS, Colwell RK, Proctor HC, Smith VS (2004). Species coextinctions and the biodiversity crisis. Science.

[4] Gerlach J (2014) *Haematopinus oliveri*. The IUCN Red List of Threatened Species 2014: e.T9621A21423551. 10.2305/IUCN.UK.2014-1.RLTS.T9621A21423551.en.

[5] Mingozzi T, Stève R (1997). Analysis of a historical extirpation of the bearded vulture *Gypaetus barbatus* (L.) in the Western Alps (France-Italy): former distribution and causes of extirpation. Biol. Conserv..

[6] Schaub M, Zink R, Beissmann H, Sarrazin F, Arlettaz R (2009). When to end releases in reintroduction programmes: demographic rates and population viability analysis of bearded vultures in the Alps. J. Appl. Ecol..

[7] BirdLife International. *Gypaetus barbatus* (amended version of 2017 assessment). The IUCN Red List of Threatened Species 2017: e.T22695174A118590506. 10.2305/IUCN.UK.2017-3.RLTS.T22695174A118590506.en, accessed 07 Apr 2021 (2017).

[8] Price, R. D., Hellenthal, R. A., Palma, R. L., Johnson, K. P., & Clayton, D. H. The chewing lice: World checklist and biological overview. Illinois Natural History Survey Special Publication 24. Illinois (2003).

[9] Clay T (1958). Revisions of mallophaga genera. *Degeeriella* from the Falconiformes. Bull. Br. Mus. (Nat. Hist.).

[10] Martín Mateo, M. P. Fauna Ibérica, Vol. 32. Phthiraptera, Ischnocera. Museo Nacional de Ciencias Naturales (CSIC), Madrid (2009).

[11] Hoberg EP, Brooks DR, Siegel-Causey D, Clayton DH, Moore J (1997). Host-parasite co-speciation: history, principles, and prospects. Host-Parasite Evolution: General Principles and Avian Models.

[12] Johnson KP, Weckstein JD, Witt CC, Faucett RC, Moyle RG (2002). The perils of using host relationships in parasite taxonomy: phylogeny of the *Degeeriella* complex. Mol. Phylogenet. Evol..

[13] Catanach TA, Johnson KP (2015). Independent origins of the feather lice (Insecta: *Degeeriella*) of raptors. Biol. J. Linn. Soc..

[14] Pérez JM, Ruiz-Martínez I, Cooper JE (1996). Occurrence of chewing lice on Spanish raptors. Ardeola.

[15] Ash JS (1960). A study of the mallophagan of birds with particular reference to their ecology. Ibis.

[16] Askew RR (1971). Parasitic Insects.

[17] Marshall AG (1981). The Ecology of Parasitic Insects.

[18] Bartlow AW, Villa SM, Thompson MW, Bush SE (2016). Walk or ride? Phoretic behaviour of amblyceran and ischnoceran lice. Int. J. Parasitol..

[19] Leonardi MS, Crespo EA, Raga JA, Fernández M (2012). Scanning electron microscopy of *Antarctophthirus microchir* (Phthiraptera: Anoplura: Echinophthiriidae): Studying morphological adaptations to aquatic life. Micron.

[20] Ortega Insaurralde I, Minoli S, Toloza AC, Picollo MI, Barrozo RB (2019). The sensory machinery of the head louse *Pediculus humanus capitis*: from the antennae to the brain. Front. Physiol..

[21] Ortega Insaurralde I, Picollo MI, Barrozo RB (2021). Sensory features of the human louse antenna: New contributions and comparisons between ecotypes. Med. Vet. Entomol..

[22] Page RDM, Lee PLM, Becher SA, Griffiths R, Clayton DH (1998). A different tempo of mitochondrial DNA evolution in birds and their parasitic lice. Mol. Phylogenet. Evol..

[23] Cruickshank RH, Johnson KP, Smith VS, Adams RJ, Clayton DH, Page RDM (2001). Phylogenetic analysis of partial sequences of elongation factor 1α identifies major groups of lice (Insecta: Phthiraptera). Mol. Phylogenet. Evol..

[24] Murrell A, Barker SC (2005). Multiple origins of parasitism in lice: phylogenetic analysis of SSU rDNA indicates that the Phthiraptera and Psocoptera are not monophyletic. Parasitol. Res..

[25] Whiteman NK, Kimball RT, Parker PG (2007). Co-phylogeography and comparative population genetics of the threatened Galápagos hawk and three ectoparasite species: ecology shapes population histories within parasite communities. Mol. Ecol..

[26] Palma RL (1978). Slide-mounting of Lice: a detailed description of the Canada Balsam technique. N. Z. Entomol..

[27] Soler-Cruz MD, Martín-Mateo MP (2009). Scanning electron microscopy of legs of two species of sucking lice (Anoplura: Phthiraptera). Micron.

[28] Hafner MS, Sudman PD, Villablanca FX, Spradling TA, Demastes JW, Nadler SA (1994). Disparate rates of molecular evolution in cospeciating hosts and parasites. Science.

[29] Simon C, Frati F, Beckenbach A, Crespi B, Liu H, Flook P (1994). Evolution, weighting and phylogenetic utility of mitochondrial gene sequences and a compilation of conserved polymerase chain reaction primers. Ann. Entomol. Soc. Am..

[30] Danforth BN, Ji S (1998). Elongation factor-1α occurs as two copies in bees: Implications for phylogenetic analysis of EF-1α sequences in insects. Mol. Biol. Evol..

[31] Smith VS, Page RDM, Johnson KP (2004). Data incongruence and the problem of avian louse phylogeny. Zool. Scr..

[32] Altschul SF, Gish W, Miller W, Myers EW, Lipman DJ (1990). Basic local alignment search tool. J. Mol. Biol..

[33] Darriba D, Taboada GL, Doallo R, Posada D (2012). jModelTest 2: More models, new heuristics and parallel computing. Nat. Methods.

[34] Huelsenbeck JP, Ronquist F (2001). MRBAYES: Bayesian inference of phylogeny. Bioinformatics.

[35] Zwickl, D. J. Genetic algorithm approaches for the phylogenetic analysis of large biological sequence datasets under the maximum likelihood criterion. Ph.D. Thesis Dissertation, The University of Texas at Austin, Texas (2006).

[36] Rambaut A (2014). FigTree v1.4.2. Institute of Evolutionary Biology.

[37] Brown CJ (1989). Plumages and measurements of the Bearded Vulture in Southern Africa. Ostrich.

[38] Chatterjee P, Payra A, Sen S, Chandra K, Gupta D, Gopi KC, Kumar V (2018). Insecta: Phthiraptera. Faunal Diversity of Indian Himalaya.

[39] Liébanas G, Sáez A, Luna A, Romero-Vidal P, Palma A, Pérez JM (2021). The morphology of *Colpocephalum pectinatum* (Phthiraptera: Amblycera: Menoponidae) under scanning electron microscopy. Arthropod Struct. Dev..

[40] Pérez, J. M. Sobre algunos aspectos de la parasitación por malógafos en aves de presa. Ph.D. thesis Dissertation, Granada University (1990).

[41] Arya G, Ahmad A, Bansal N, Saxena R, Saxena AK (2010). Nature of placodean sensilla of four ischnoceran Phthiraptera. Entomon.

[42] Khan V, Bansal N, Arya G, Ahmad A, Saxena AK (2011). Contribution to the morphology of *Degeeriella regalis* (Insecta, Phthiraptera, Ischnocera). J. Entomol. Res..

[43] Agarwal GP, Ahmad A, Rashmi A, Arya G, Bansal N, Saxena AK (2011). Bio-ecology of the louse, *Upupicola upupae*, infesting the Common Hoopoe, *Upupa epops*. J. Insect. Sci..

[44] Singh P, Gupta N, Khan G, Kumar S, Ahmad A (2019). Diagnostic characters of three nymphal instars and morphological features of adult Collard-dove louse *Columbicola bacillus* (Phthiraptera: Insecta). J. Appl. Nat. Sci..

[45] Clayton DH, Johnson KP (2003). Linking coevolutionary history to ecological process: Doves and lice. Evolution.

[46] Barker SC (1996). Lice, cospeciation and parasitism. Int J Parasitol.

[47] Page RDM, Clayton DH, Paterson AA (1996). Lice and cospeciation: A response to Barker. Int. J. Parasitol..

[48] Paterson AM, Gray RD, Clayton DH, Moore J (1997). Host-parasite cospeciation, host-switching and missing the boat. Host-Parasite Evolution: General Principles and Avian Models.

[49] Paterson AM, Palma RL, Gray RD (1999). How frequently do avian lice miss the boat? Implications for coevolutionary studies. Syst. Biol..

[50] Frey H, Walter W, Meyburg BU, Chancellor RD (1989). The reintroduction of the bearded vulture *Gypaetus barbatus* into the Alps. Raptors in the Modern World.

[51] Pérez JM, Sánchez I, Palma RL (2013). The dilemma of conserving parasites: the case of *Felicola (Lorisicola) isidoroi* (Phthiraptera: Trichodectidae) and its host, the endangered Iberian lynx (*Lynx pardinus*). Insect. Conserv. Divers..

